# Associations between composite dietary antioxidant index and depressive symptoms among pregnant and postpartum women

**DOI:** 10.1186/s12884-026-09047-8

**Published:** 2026-04-10

**Authors:** LvJiao Zhang, Ying Huang, Yannv Qu, Xiaochen Ma

**Affiliations:** 1 Department of Obstetrics & Gynecology, Jinan Maternity and Child Care Hospital, Jinan, 250022 China; 2 Department of Geriatrics, Peking University Shenzhen Hospital, Shenzhen Peking University, The Hong Kong University of Science and Technology Medical Center, Shenzhen, 518036 China

**Keywords:** Composite Dietary Antioxidant Index, Depression, Pregnancy, Postpartum, Perinatal

## Abstract

**Background:**

Perinatal depression is a prevalent mood disorder associated with nutritional factors, yet the relationship between comprehensive dietary antioxidant capacity and perinatal depression remains unclear. This study aimed to investigate the association between the Composite Dietary Antioxidant Index (CDAI) and depressive symptoms in a sample of U.S. perinatal women.

**Methods:**

We analyzed a nationally representative sample of 1,093 pregnant and postpartum (within 18 months) women from NHANES 2005–2018. The CDAI, a composite metric integrating six key antioxidant nutrients (vitamins A, C, E, zinc, selenium, carotenoids), was derived from 24-hour dietary recalls. Depressive symptoms were quantified using the PHQ-9, with a score ≥ 10 defining clinically significant depression. We employed segmented regression modeling to identify a potential inflection point in the CDAI-depression relationship, moving beyond the constraints of linear assumptions. The association was evaluated comprehensively using both linear (for symptom severity) and logistic (for clinical caseness) models. Robustness was assessed through extensive sensitivity analyses, and we further explored effect modification by key lifestyle factors.

**Results:**

We observed a significant nonlinear association between the CDAI and depressive symptoms. Below the identified inflection point (CDAI ≤ − 2.75), indicative of suboptimal antioxidant status, each unit increase in CDAI was significantly associated with both a 0.80-point reduction in PHQ-9 score (β = −0.80, 95% CI: −1.22, − 0.38; *P* = 0.0002) and a 39% lower odds of clinical depression (OR = 0.61, 95% CI: 0.45, 0.83; *P* = 0.0019). This protective association was most potent in vulnerable subgroups, including never-smokers (*P*-interaction = 0.0007) and Non-Hispanic Black women (*P*-interaction = 0.014).

**Conclusion:**

In this cross-sectional study, we observed a significant nonlinear association between the CDAI and depressive symptoms among U.S. perinatal women. Lower dietary antioxidant capacity was linked to a significantly increased severity of depressive symptoms and higher odds of clinical depression, whereas higher antioxidant intake beyond the threshold did not yield further incremental benefits.

**Supplementary Information:**

The online version contains supplementary material available at 10.1186/s12884-026-09047-8.

## Introduction

Perinatal depression, encompassing major depressive episodes during pregnancy or within the first year postpartum, is a common and potentially severe mood disorder. It is estimated to affect roughly 1 in 7 women during the perinatal period [1]. Globally, about 10% of pregnant women and 13% of postpartum women experience a depressive disorder, with higher rates reported in many low-income settings [2]. In the United States, the estimated incidence of postpartum major depressive disorder ranges from 8.9% in pregnant women to 37% at any time in the first postpartum year [3]. Perinatal depression is more enduring and impairing than the transient “baby blues”, often manifesting as persistent sadness, anxiety, irritability, sleep/appetite disturbances, and feelings of guilt or hopelessness [4–6]. If left untreated, it can significantly interfere with a mother’s ability to care for herself and her infant, and is associated with adverse outcomes such as impaired mother-infant bonding, developmental delays in children, and in severe cases maternal suicidality [5]. Despite its impact, up to 50% of perinatal depression cases may go undiagnosed or untreated. There is a pressing need for preventive strategies and modifiable risk factor identification to support maternal mental health.

Among the various biological and psychosocial factors implicated in perinatal depression, nutrition has emerged as an important area of research [7]. Pregnancy and the postpartum period impose increased nutritional demands and are characterized by heightened oxidative stress and inflammatory activity [8]. Oxidative stress—an imbalance between pro-oxidants and antioxidants in the body—can contribute to neuronal damage, apoptosis, and inflammation in the brain, which are thought to play a role in the pathophysiology of depression [9, 10]. Notably, lower levels of antioxidants in plasma have been associated with the development of postpartum depression (PPD) [8]. These observations raise the question of whether diets rich in antioxidants might protect against perinatal depression by counteracting oxidative stress. Antioxidants obtained from diet (such as vitamins and minerals) help neutralize reactive oxygen species and reduce systemic inflammation, potentially exerting neuroprotective effects [11]. Prior studies in non-perinatal populations have linked higher consumption of antioxidant-rich foods to better mental health [12]. For example, older adults with depression have been found to consume fewer fruits, vegetables, and antioxidants than their non-depressed peers [13]. In general adult samples, greater intake of specific antioxidant nutrients―including vitamin C, vitamin E, vitamin A (beta-carotene), zinc, and selenium―has been associated with lower odds of depression or depressive symptoms [14–17]. Meta-analyses of observational studies report that individuals with higher dietary vitamin C and E intake have a significantly reduced risk of depression compared to those with low intake. Furthermore, clinical research suggests a therapeutic potential of antioxidants; for instance, supplementation with antioxidant vitamin E has shown beneficial effects as an adjunct treatment for depression and anxiety disorders. In the context of perinatal mental health, emerging evidence supports the link between diet quality and depression. A prospective cohort study in Greece found that pregnant women who adhered to a “health-conscious” diet (rich in vegetables, fruits, legumes, nuts, fish, and olive oil) had approximately 50% lower risk of postpartum depression compared to those with less healthy dietary patterns [18]. Similarly, a recent study in Iran demonstrated that women with higher overall antioxidant/pro-oxidant balance scores during pregnancy were significantly less likely to develop postpartum depression [8]. Diets abundant in fruits and vegetables (which are high in antioxidant vitamins) and adequate selenium intake have each been associated with a lower risk of perinatal depressive symptoms. These findings collectively suggest that nutritional antioxidant status may be an important, modifiable factor in perinatal depression.

The Composite Dietary Antioxidant Index (CDAI) is a comprehensive metric designed to quantify the antioxidant capacity of an individual’s diet. Developed by Wright et al. the CDAI combines intake levels of multiple dietary antioxidants into a single score [19]. In its typical formulation, CDAI is calculated by summing the standardized intakes of several vitamins and minerals with antioxidant properties―commonly vitamin A, vitamin C, vitamin E, zinc, selenium, and carotenoids [20]―for each person. This yields a composite score where higher values indicate a diet with greater overall antioxidant potential. Initially, CDAI was applied in cancer epidemiology to examine diet and cancer risk [21], but more recently it has been utilized in studies of metabolic and mental health outcomes. For example, Zhao et al. investigated CDAI in a general U.S. adult population and found that higher CDAI was associated with lower prevalence of depression in a nonlinear fashion. In that study, the odds of depression decreased by about 30% for each unit increase in CDAI up to an inflection point around CDAI = 0.16, after which the association plateaued. However, data are very limited on CDAI and mental health specifically in pregnant and postpartum women. Given the unique physiological stresses of the perinatal period and the potential importance of diet during this time, it is worthwhile to explore whether the relationship between dietary antioxidants and depression observed in the general population holds true for expecting and new mothers.

In this study, we aimed to examine the association between CDAI and depressive symptoms among pregnant and postpartum women in the United States, using data from NHANES (2005–2018). We hypothesized that a higher CDAI (indicating greater dietary antioxidant intake) would be associated with lower depressive symptom scores in this population. We also explored the possibility of a nonlinear (threshold or saturation) relationship.

## Methods

### Study design and population

We analyzed data from NHANES for the years 2005 through 2018. NHANES is an ongoing series of cross-sectional surveys conducted by the National Center for Health Statistics, using a complex multistage probability sample to assess the health and nutritional status of the civilian, non-institutionalized U.S. population. Each NHANES cycle involves in-home interviews, standardized physical examinations, laboratory tests, and dietary assessments for thousands of participants, and all participants provide informed consent under protocols approved by institutional review boards. For the present study, we combined data from multiple two-year NHANES cycles (2005–06, 2007–08, …, 2017–18) to obtain a larger sample of women in the perinatal period.

**Fig. 1:**
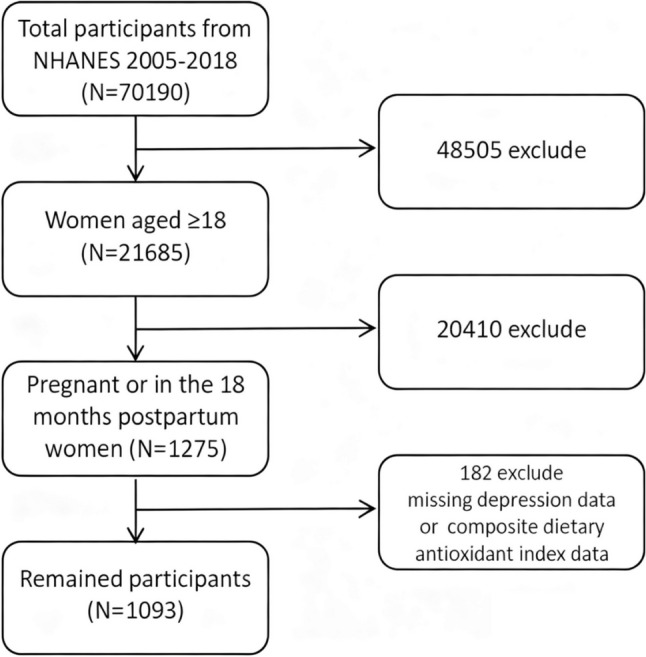
Flow chart of participant selection from the NHANES 2005–2018

We included women of reproductive age who were either pregnant or within 18 months postpartum at the time of their NHANES participation. In NHANES, pregnancy status was ascertained through a combination of self-report and laboratory testing (urine pregnancy test) during the physical exam. We considered a participant as postpartum if she reported having delivered a live infant within the past 18 months prior to the survey. We excluded women younger than 18 years (minors) and those older than 50 years to focus on typical childbearing ages. From the pooled 2005–2018 data, a total of 1,275 women were identified as pregnant or postpartum. We further excluded individuals with missing data on key variables: 24-hour dietary recall data needed for calculating CDAI or PHQ-9 depression questionnaire data. Our final analytic sample consisted of 1,093 perinatal women with complete information on diet and depressive symptoms (Fig. 1).

### Dietary assessment and CDAI calculation

Dietary intake was evaluated in NHANES via 24-hour dietary recall interviews administered by trained interviewers. In most cycles, each participant completed two 24-hour recalls: one in-person recall at the Mobile Examination Center and a second recall via telephone 3–10 days later. The recalls collected detailed information on all foods and beverages consumed from midnight to midnight of the day prior to the interview. Nutrient intakes were then estimated using the USDA Food and Nutrient Database for Dietary Studies. Because the validity of 24-hour dietary recalls for generating composite measures is heavily influenced by the number of recalls, all participants in our analytic sample completed two 24-hour dietary recalls. We calculated the CDAI for each participant using the average intake of the six antioxidant nutrients across the two recall days [22, 23]. To improve transparency regarding the reliability of the dietary exposure assessment, we reported the percentage of participants whose CDAI was calculated using only one dietary recall for both the overall NHANES population and our analytic sample (Table S1).

We focused on six antioxidant-related nutrients that are components of the Composite Dietary Antioxidant Index (CDAI): vitamin A, vitamin C, vitamin E, zinc (Zn), selenium (Se), and carotenoids [20]. These nutrients were selected based on prior literature for their antioxidant properties and roles in redox balance. Following the method originally described by Wright et al. [19], we calculated the CDAI for each individual by standardizing and summing the intakes of these nutrients. Specifically, for each of the six nutrients, we computed a Z-score = (individual’s intake—mean intake)/standard deviation, using the means and SDs from our analytic sample. The CDAI was then defined as the sum of the six nutrient-specific Z-scores. Thus, CDAI is a continuous variable; higher CDAI values indicate a diet with higher overall antioxidant nutrient content relative to the sample, whereas lower CDAI values indicate poorer antioxidant intake.

### Depressive symptom assessment

The outcome of interest was depressive symptom severity, measured by the Patient Health Questionnaire-9 (PHQ-9). The PHQ-9 is a well-validated self-report instrument that screens for depression by asking respondents how often in the past two weeks they have experienced each of 9 symptoms (derived from DSM-IV criteria for major depression), such as depressed mood, anhedonia, sleep disturbances, fatigue, appetite change, feelings of worthlessness, concentration difficulty, psychomotor changes, and suicidal thoughts [24]. Each item is rated on a frequency scale from 0 (“not at all”) to 3 (“nearly every day”), yielding a total score. Higher scores indicate more severe depressive symptoms. The PHQ-9 ranges from 0 to 27, with 0–4 indicating no depression and ≥ 5 suggesting depressive symptoms. Severity is further categorized as mild (5–9), moderate (10–14), or severe (15–27). The PHQ-9 has been widely used in perinatal populations and performs similarly to specialized scales like the Edinburgh Postnatal Depression Scale for detecting perinatal depression [25]. In NHANES, the PHQ-9 questionnaire was included for participants aged 18 and older in the mobile examination center interview.

For our analysis, we treated the PHQ-9 total score as a continuous outcome in primary analyses, providing a measure of depressive symptom burden. Additionally, we created a binary variable of possible major depression defined as PHQ-9 score ≥ 10, which is a standard cutoff indicating moderate-to-severe depressive symptoms and has good sensitivity and specificity for major depressive disorder diagnosis in clinical validation studies. This binary outcome was used in secondary analyses (logistic regression). In addition, we analyzed PHQ-9 ≥ 5 and PHQ-9 ≥ 15 as different cutoff points in sensitivity analysis.

### Covariates

We included a range of covariates in our regression models to control for potential confounding factors. Covariate selection was guided by prior literature on depression and diet as well as \ability in NHANES. The following variables were included as adjustment covariates: age (years), race/ethnicity (Non-Hispanic White, Non-Hispanic Black, Hispanic, and Other), poverty-to-income ratio, marital status, body mass index (BMI), white blood cell count (×10^9/L), smoking status, and alcohol use. Detailed measurement procedures can be found at https://www.cdc.gov/nchs/nhanes, accessed on September 8, 2025.

### Statistical analysis

We first conducted descriptive analyses to characterize the study sample. We calculated means and standard deviations for continuous variables and proportions for categorical variables. We summarized the distribution of CDAI and PHQ-9 scores in the sample, as well as the prevalence of PHQ-9 ≥ 10.

For the primary analytical objective, we used multivariable linear regression to examine the association between CDAI (independent variable) and PHQ-9 score (dependent variable), adjusting for the covariates listed above. Given evidence from previous studies that the relationship might not be strictly linear, we conducted piecewise linear (two-segment) regression to assess potential nonlinearity in the CDAI–depression association. Based on the results and using methods described by Zhao et al. [21], we implemented a segmented regression (also known as a two-piecewise linear regression or threshold regression). This involved identifying a potential inflection point or threshold in CDAI at which the slope of the association with PHQ-9 changes. The value of CDAI at this inflection point was then used to define two segments: a “low CDAI” segment (below the threshold) and a “high CDAI” segment (above the threshold). This yields separate slope estimates for the association between CDAI and PHQ-9 in the two ranges, while ensuring continuity at the join point. We again used a likelihood ratio test to compare this two-piecewise linear model against a single linear model to confirm whether adding the inflection point significantly improved fit. The two-piecewise model provides interpretable estimates of β in each range of CDAI.

For the secondary analysis, we ran logistic regression models with depression (PHQ-9 score ≥ 10) as the outcome. We mirrored the approach above: first, linear vs. nonlinear fit was assessed, and then a piecewise linear model with the same threshold (CDAI − 2.75) was used for consistency.

In sensitivity analyses, we used multivariable logistic regression to model the odds of PHQ-9 ≥ 5 (mild symptoms) and PHQ-9 ≥ 15 (severe depression). Nonlinearity was assessed using piecewise logistic regression, consistent with the primary approach. This allowed us to examine if the association with CDAI was robust across different depression definitions. Results are presented as odds ratios (ORs) per unit change in CDAI for each segment of the piecewise model.

We further performed stratified analyses to see whether the relationship between CDAI-depression was consistent across subgroups and whether there was an interaction. In particular, we stratified by age group (18–30 and 30–47 years), BMI group (15.6–25 and 25–73.5), WBC group (2.5–10 and 10–20), race, marital status, poverty income ratio, education level, smoking status, pregnant, and alcohol use to explore any effect modification.

We note that NHANES uses a complex sampling design with sample weights, stratification, and clustering. However, for this exploratory analysis our focus was on the association between variables rather than producing nationally representative prevalence estimates. Therefore, we did not apply sample weights in the primary models. This approach yields internally valid estimates of associations but may not generalize prevalence or mean levels to the U.S. population. All analyses were conducted using EmpowerStats (http://www.empowerstats.com) and R 4.2.2. Statistical significance was determined at the two-tailed α = 0.05 level.

## Results

### Participant characteristics

A total of 1,093 women in the perinatal period (pregnancy or within 18 months postpartum) were included in this analysis. Key demographic and clinical characteristics of the sample are summarized here (Table 1). The mean age was 28.36 years, with a standard deviation of 5.73 years (range approximately 18 to 47 years). The racial/ethnic composition was diverse: roughly 38% identified as non-Hispanic White, 17% as non-Hispanic Black, 24% as Mexican American, 9% as Hispanic, and the remainder as other ethnicities. Most of the women (about 79%) were married or living with a partner.

**Table 1:** The demographic and clinical characteristics of the participants

Characteristics	Total	CDAI tertile			*P*-value
		Low (−7.37- −0.48)	Middle (−0.48-2.58)	High (2.58–20.00.58.00)	
Participants	1093	364	364	365	
Age (years)	28.36 ± 5.73	27.90 ± 5.87	28.31 ± 5.73	28.87 ± 5.54	0.027
BMI (kg/m^2^)	29.38 ± 7.01	29.48 ± 7.20	30.02 ± 7.07	28.64 ± 6.69	0.027
WBC (1000 cells/µL)	8.46 ± 2.52	8.14 ± 2.31	8.62 ± 2.61	8.60 ± 2.59	0.018
PHQ-9 total score	3.35 ± 3.84	3.59 ± 4.19	3.17 ± 3.47	3.29 ± 3.81	0.306
CDAI	1.53 ± 4.03	−2.43 ± 1.46	1.05 ± 0.87	5.98 ± 3.33	< 0.001
Race/ethnicity (%)					0.105
Non-Hispanic White	418 (38.24%)	125 (34.34%)	153 (42.03%)	140 (38.36%)	
Non-Hispanic Black	191 (17.47%)	69 (18.96%)	67 (18.41%)	55 (15.07%)	
Mexican American	262 (23.97%)	91 (25.00%)	76 (20.88%)	95 (26.03%)	
Other Hispanic	103 (9.42%)	44 (12.09%)	31 (8.52%)	28 (7.67%)	
Other Race	119 (10.89%)	35 (9.62%)	37 (10.16%)	47 (12.88%)	
Marital status (%)					0.189
Married/Living with Partner	859 (78.59%)	271 (74.45%)	291 (79.95%)	297 (81.37%)	
Widowed/Divorced/Separated	50 (4.57%)	20 (5.49%)	14 (3.85%)	16 (4.38%)	
Never married	184 (16.83%)	73 (20.05%)	59 (16.21%)	52 (14.25%)	
Poverty income ratio (%)					< 0.001
Poor	318 (29.09%)	136 (37.36%)	99 (27.20%)	83 (22.74%)	
Nearly poor	266 (24.34%)	89 (24.45%)	90 (24.73%)	87 (23.84%)	
Middle income	237 (21.68%)	72 (19.78%)	75 (20.60%)	90 (24.66%)	
High income	189 (17.29%)	39 (10.71%)	71 (19.51%)	79 (21.64%)	
Missing	83 (7.59%)	28 (7.69%)	29 (7.97%)	26 (7.12%)	
Education level (%)					< 0.001
Below high school	71 (6.50%)	34 (9.34%)	21 (5.77%)	16 (4.38%)	
High school	420 (38.43%)	163 (44.78%)	138 (37.91%)	119 (32.60%)	
Above high school	602 (55.08%)	167 (45.88%)	205 (56.32%)	230 (63.01%)	
Smoking status (%)					< 0.001
Never	764 (69.90%)	248 (68.13%)	252 (69.23%)	264 (72.33%)	
Former	152 (13.91%)	35 (9.62%)	55 (15.11%)	62 (16.99%)	
Now	155 (14.18%)	73 (20.05%)	50 (13.74%)	32 (8.77%)	
Missing	22 (2.01%)	8 (2.20%)	7 (1.92%)	7 (1.92%)	
Depression (%)					0.107
No	1011 (92.50%)	329 (90.38%)	344 (94.51%)	338 (92.60%)	
Yes	82 (7.50%)	35 (9.62%)	20 (5.49%)	27 (7.40%)	
Hypertension (%)					0.407
No	980 (89.66%)	321 (88.19%)	332 (91.21%)	327 (89.59%)	
Yes	113 (10.34%)	43 (11.81%)	32 (8.79%)	38 (10.41%)	
Diabetes Mellitus (%)					0.831
No	526 (91.80%)	218 (91.21%)	161 (94.15%)	147 (90.18%)	
Yes	18 (3.14%)	9 (3.77%)	4 (2.34%)	5 (3.07%)	
IFG	12 (2.09%)	5 (2.09%)	3 (1.75%)	4 (2.45%)	
IGT	17 (2.97%)	7 (2.93%)	3 (1.75%)	7 (4.29%)	
Hyperlipidemia (%)					0.239
No	383 (35.04%)	129 (35.44%)	116 (31.87%)	138 (37.81%)	
Yes	710 (64.96%)	235 (64.56%)	248 (68.13%)	227 (62.19%)	
Pregnant (%)					< 0.001
No	563 (52.76%)	232 (65.91%)	170 (48.02%)	161 (44.60%)	
Yes	504 (47.24%)	120 (34.09%)	184 (51.98%)	200 (55.40%)	
Alcohol use (%)					0.084
Never	231 (21.13%)	95 (26.10%)	67 (18.41%)	69 (18.90%)	
Former	201 (18.39%)	65 (17.86%)	64 (17.58%)	72 (19.73%)	
Mild	216 (19.76%)	60 (16.48%)	74 (20.33%)	82 (22.47%)	
Moderate	195 (17.84%)	54 (14.84%)	73 (20.05%)	68 (18.63%)	
Heavy	202 (18.48%)	70 (19.23%)	73 (20.05%)	59 (16.16%)	
Missing	48 (4.39%)	20 (5.49%)	13 (3.57%)	15 (4.11%)	

Mean ± SD for continuous variables. Number (%) for categorical variables

*BMI* Body mass index, *WBC* White Blood Cell, *CDAI* Composite dietary antioxidant index, *IFG* Impaired Fasting Glycaemia, *IGT* Impaired Glucose Tolerance

In terms of mental health, the overall mean PHQ-9 score was 3.35 (SD 3.84) on a 0–27 scale, indicating that, on average, women reported relatively low levels of depressive symptoms, and 82 women (7.5% of the sample) had PHQ-9 scores of 10 or higher, consistent with at least moderate depressive symptoms and considered a positive screen for possible major depression. And 24 women (2.2% of the sample) had PHQ-9 scores ≥ 15, indicating moderately severe to severe symptoms.

Regarding dietary antioxidant intake, the Composite Dietary Antioxidant Index (CDAI) in our sample had a median of 1.09, with a mean around 1.53 and a standard deviation of approximately 4.03 (due to summing six Z-scored nutrients). The minimum CDAI observed was about − 7.37 and the maximum was about + 20.00, indicating some individuals with very low or very high antioxidant diets. A CDAI of zero corresponds to an average antioxidant intake (for each nutrient) equivalent to the sample mean.

There were some notable differences in characteristics across the range of CDAI. Women with higher CDAI (in the high tertile) were more likely to be older, non-smokers, and have higher PIR (suggesting higher socioeconomic status) compared to those with low CDAI, consistent with known correlates of healthier diets. Importantly, we observed a crude trend where mean PHQ-9 scores were highest in the low CDAI tertile (mean PHQ-9 ~ 3.59).

### Univariate analysis

To identify the covariates for the association of CDAI with PHQ-9 total scores, we applied univariate linear regression analyses separately. Age, race, marital status, poverty income ratio, smoking status, BMI, hypertension, hyperlipidemia, and alcohol use were associated with PHQ-9 total scores (all *P* < 0.05) (Table 2).

**Table 2:** Correlations of clinical characteristics for PHQ-9 total scores

Characteristics	β (95% CI)	*P*-value
Age (years)	−0.07 (−0.11, −0.03)	0.0005
Race/ethnicity		
Non-Hispanic White	Ref.	
Non-Hispanic Black	0.54 (−0.11, 1.20)	0.1058
Mexican American	0.07 (−0.52, 0.67)	0.8051
Other Hispanic	−0.23 (−1.06, 0.59)	0.5802
Other Race	−0.81 (−1.59, −0.03)	0.0414
Marital status		
Married/Living with Partner	Ref.	
Widowed/Divorced/Separated	1.56 (0.47, 2.65)	0.0049
Never married	1.07 (0.46, 1.67)	0.0006
Poverty income ratio		
Poor	Ref.	
Nearly poor	−0.35 (−0.97, 0.27)	0.2650
Middle income	−0.72 (−1.36, −0.08)	0.0276
High income	−1.53 (−2.21, −0.84)	< 0.0001
Missing	0.31 (−0.61, 1.23)	0.5065
Education level		
Below high school	Ref.	
High school	0.05 (−0.91, 1.01)	0.9137
Above high school	−0.70 (−1.64, 0.24)	0.1426
Smoking status		
Never	Ref.	
Former	0.51 (−0.15, 1.16)	0.1297
Now	2.12 (1.47, 2.77)	< 0.0001
Missing	1.04 (−0.56, 2.64)	0.2020
BMI (kg/m^2^)	0.07 (0.04, 0.10)	< 0.0001
WBC (1000 cells/µL)	0.05 (−0.04, 0.15)	0.2666
Hypertension		
No	Ref.	
Yes	2.22 (1.49, 2.96)	< 0.0001
Diabetes Mellitus		
No	Ref.	
Yes	−0.34 (−2.24, 1.56)	0.7259
IFG	−2.09 (−4.41, 0.23)	0.0774
IGT	0.31 (−1.65, 2.26)	0.7585
Hyperlipidemia		
No	Ref.	
Yes	0.52 (0.04, 1.00)	0.0322
Pregnant		
No	Ref.	
Yes	0.07 (−0.39, 0.53)	0.7614
CDAI	−0.04 (−0.10, 0.02)	0.1549
Alcohol use		
Never	Ref.	
Former	0.27 (−0.45, 0.99)	0.4599
Mild	−0.17 (−0.88, 0.54)	0.6345
Moderate	0.82 (0.09, 1.55)	0.0277
Heavy	0.96 (0.24, 1.69)	0.0089
Missing	0.45 (−0.74, 1.63)	0.4616

*BMI* Body mass index, *WBC* White Blood Cell, *CDAI* Composite dietary antioxidant index, *IFG* Impaired Fasting Glycaemia, *IGT* Impaired Glucose Tolerance

### Association between CDAI and depressive symptoms severity

**Fig. 2:**
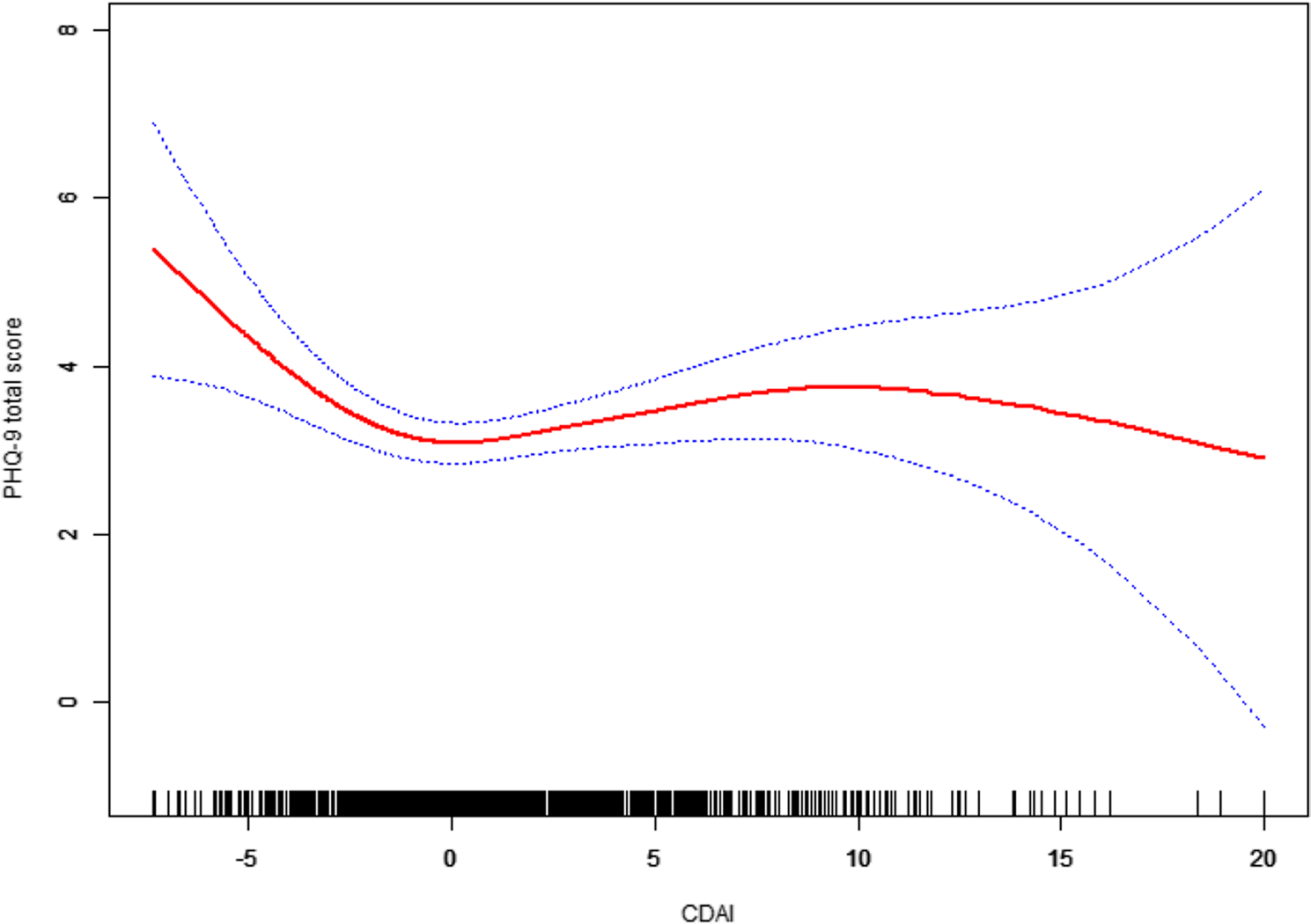
The association between Composite dietary antioxidant index and PHQ-9 total scores. Models were adjusted for age, ethnicity, poverty income ratio, marital status, white blood cell count, body mass index, smoking status, and alcohol use

In multivariable regression analyses with smoothing spline fitting, adjusted for all covariates (age, race/ethnicity, PIR, marital status, BMI, WBC, smoking, alcohol), we found the association between CDAI and PHQ-9 depressive symptoms was nonlinear. We further observed a saturation effect between the CDAI and depression when conducting smoothing curve fitting in the fully adjusted model (Fig. 2).

To characterize this relationship more concretely, we implemented a two-piecewise linear regression with one inflection point (Table 3). The algorithm identified CDAI = − 2.75 as the optimal inflection point (i.e., the point where the slope change yielded the best model fit). In the two-piecewise linear model, we observed a marked difference in association between the two segments:

**Table 3:** Threshold effect analysis of composite dietary antioxidant index on PHQ-9 total score

CDAI	β (95% CI)	*P*-value
Fitting by standard linear model	−0.01 (−0.06, 0.05)	0.8633
Fitting by two-piecewise linear model		
Inflection point	−2.75	
≤ −2.75	−0.80 (−1.22, −0.38)	0.0002
> −2.75	0.05 (−0.02, 0.11)	0.1607
Log-likelihood ratio	< 0.001	

Models were adjusted for age, ethnicity, poverty income ratio, marital status, white blood cell count, body mass index, smoking status, and alcohol use

Low CDAI segment (CDAI ≤–2.75): In this range, higher CDAI was strongly associated with lower PHQ-9 scores. The β coefficient was − 0.80 for the association between CDAI and PHQ-9 (95% CI − 1.22, − 0.38; *P* = 0.0002). This means that for women with very low antioxidant diets, each 1.0 increase in CDAI (which could result, for example, from adding a couple of servings of fruits/vegetables or equivalent nutrient intake improvements) was associated with a reduction of 0.8 points in the PHQ-9 score, on average, holding other factors constant.

High CDAI segment (CDAI >–2.75): In contrast, for women with CDAI in the higher range (from − 2.75 up to ~ + 20), each one-point increase in CDAI was associated with an increase in PHQ-9 score (β = 0.05, 95% CI − 0.02, 0.11), but the association was null (*P* = 0.16).

The log-likelihood ratio test comparing this two-piecewise model to a single-slope linear model was highly significant (*P* < 0.001), confirming that the two-piecewise model provided a substantially better fit to the data than a one-piece linear model. This provides strong evidence of a threshold/saturation effect in the relationship between dietary antioxidant index and depressive symptoms.

### Association between CDAI and depression

**Fig. 3:**
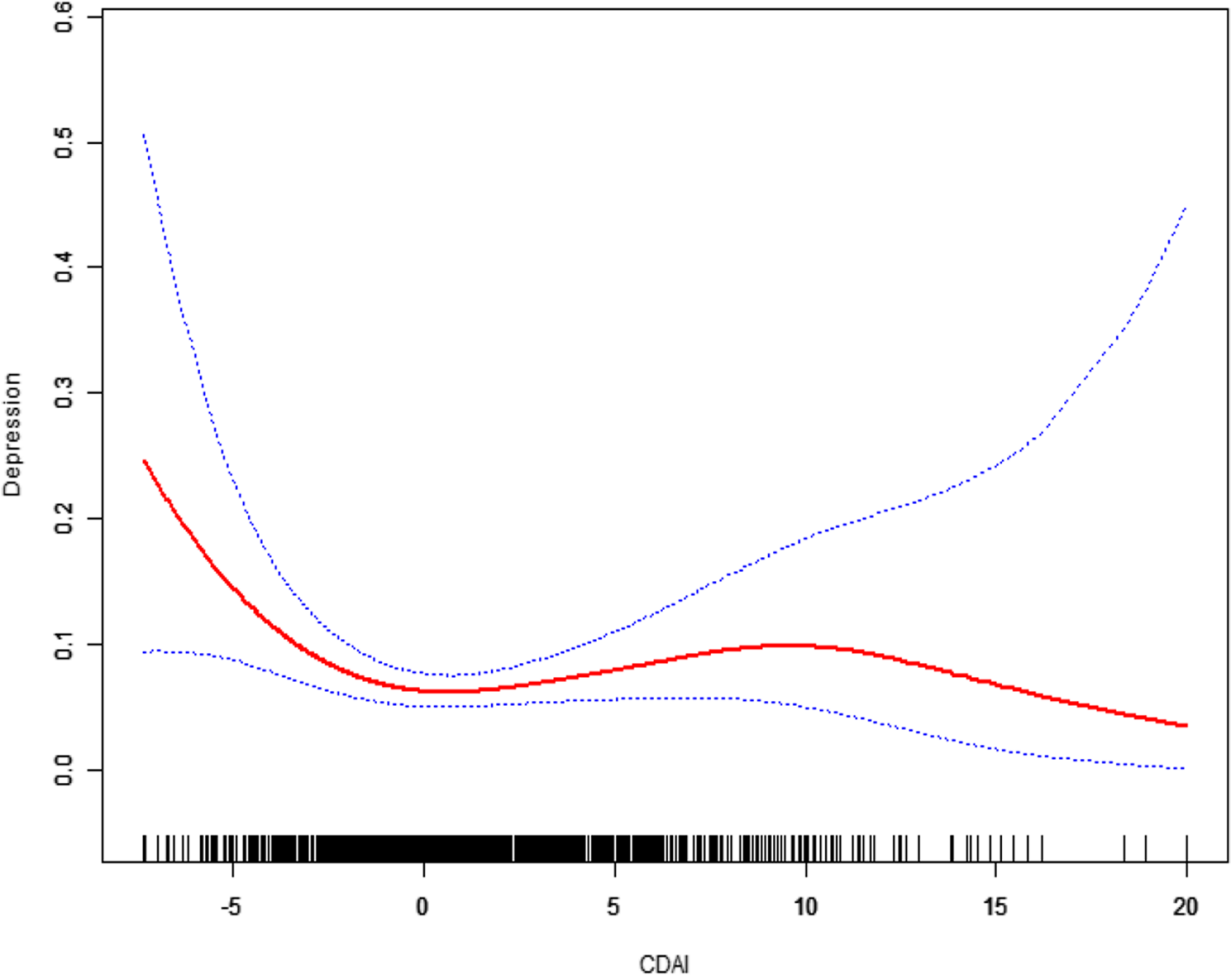
The association between Composite dietary antioxidant index and Depression (PHQ-9 total score ≥ 10). Models were adjusted for age, ethnicity, poverty income ratio, marital status, white blood cell count, body mass index, smoking status, and alcohol use

When depressive symptoms were defined as a binary outcome (PHQ-9 score ≥ 10), a similar nonlinear pattern was observed after full adjustment (Fig. 3), suggesting the presence of a potential turning point.

In the adjusted logistic regression with a threshold, we found the following (Table 4):

**Table 4:** Threshold effect analysis of composite dietary antioxidant index on depression (PHQ-9 ≥ 10)

CDAI	OR (95% CI)	*P*-value
Fitting by standard linear model	0.97 (0.91, 1.04)	0.4156
Fitting by two-piecewise linear model		
Inflection point	−2.75	
≤ −2.75	0.61 (0.45, 0.83)	0.0019
> −2.75	1.02 (0.96, 1.09)	0.4877
Log-likelihood ratio	0.004	

Models were adjusted for age, ethnicity, poverty income ratio, marital status, white blood cell count, body mass index, smoking status, and alcohol use

For CDAI ≤ − 2.75, a significant negative association was observed between the CDAI and depression (OR = 0.61, 95% CI: 0.45, 0.83; *P* = 0.0019). Within this range, each unit increase in CDAI was associated with a nearly 40% reduction in the odds of having depression.

For CDAI > − 2.75, no significant association was found between CDAI and depression (OR = 1.02, 95% CI: 0.96, 1.09; *P* = 0.4877). This indicates that increasing CDAI levels beyond this threshold were not linked to a statistically significant change in the odds of depression.

### Sensitivity analyses

When we treated depressive symptoms as dichotomous outcomes (defined as PHQ-9 ≥ 5 and ≥ 15) and conducted smoothing curve fitting in the fully adjusted model, Figure S1 and Figure S2 also show a nonlinear relationship. We observed a significant inverse association between CDAI and the odds of depressive symptoms among pregnant and postpartum women. Specifically, using piecewise logistic models with the previously identified threshold (− 2.75), we found similar results (Table S2, Table S3).

For CDAI values below the inflection point (≤ − 2.75), each 1-unit increase was associated with significantly lower odds of depression, with an OR = 0.77 (95% CI: 0.61, 0.99; *P* = 0.0406) for a PHQ-9 ≥ 5 and an OR = 0.49 (95% CI: 0.30, 0.79; *P* = 0.0033) for a PHQ-9 ≥ 15. This indicates that in the low-antioxidant group, higher CDAI was linked to substantially lower odds of being depressed. For CDAI above the inflection point (> − 2.75), no statistically significant associations were observed for either depressive symptoms definition (PHQ-9 ≥ 5 or ≥ 15; all *P* > 0.05). Likelihood ratio tests again supported the threshold models over a linear model (*P* = 0.05 for PHQ-9 ≥ 5 and *P* = 0.006 for PHQ-9 ≥ 15). These results reinforce that the relationship between antioxidant diet and clinically relevant depression is present primarily at the lower end of the intake spectrum.

Considering that C-reactive protein (CRP) is a more robust measure of chronic inflammation and has been linked to depression in population-based studies, we additionally adjusted for CRP as a covariate in sensitivity analyses. Despite the substantial reduction in sample size (*n* = 257), smoothing curve fitting revealed nonlinear patterns between CDAI and both PHQ-9 score and depression that were visually consistent with our main findings (Figure S3, Figure S4).

### Stratified analyses

**Fig. 4:**
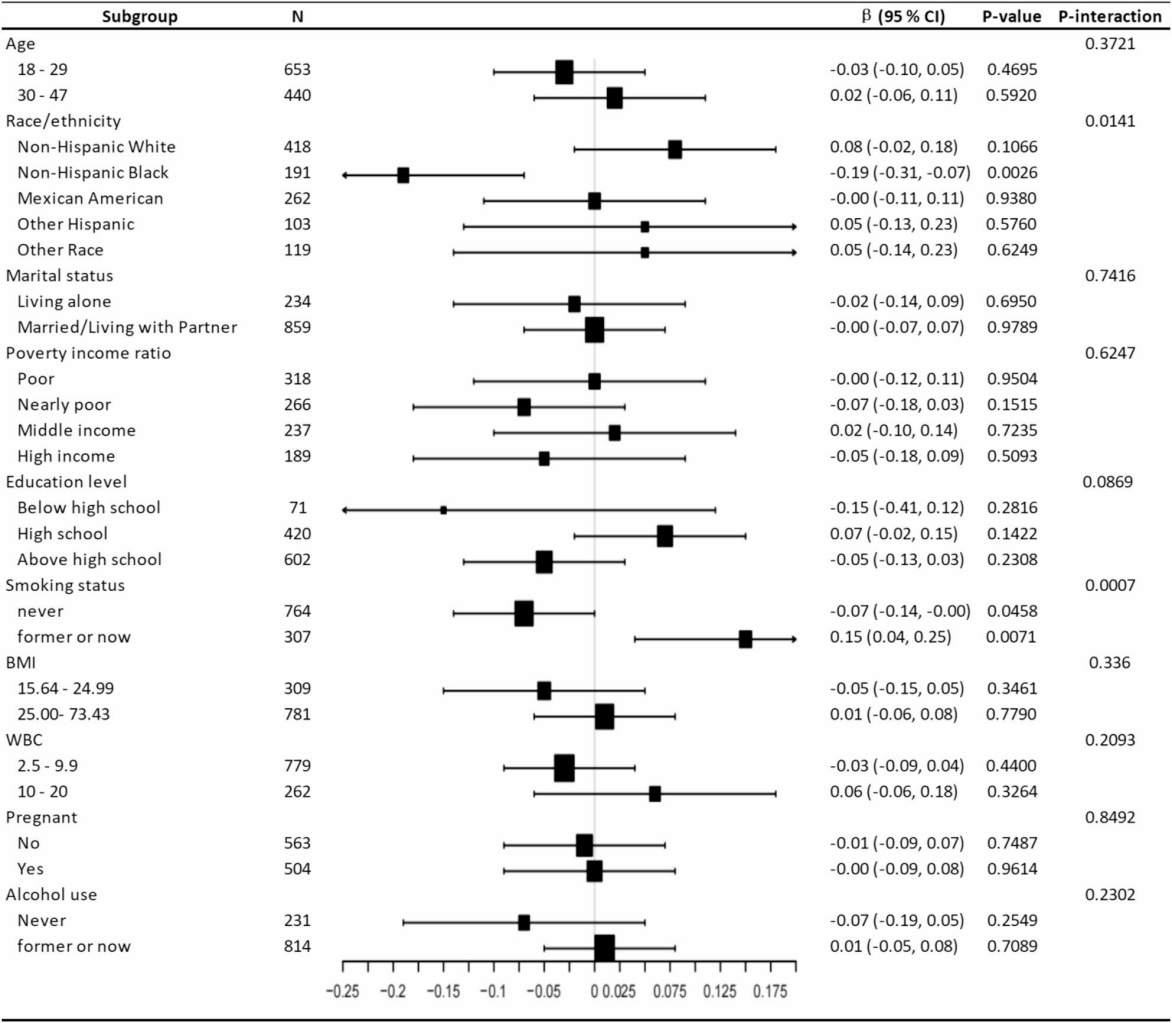
Subgroup analysis of the relationship between Composite dietary antioxidant index and PHQ-9 total score. Age, ethnicity, poverty income ratio, marital status, white blood cell count, body mass index, smoking status, alcohol use were adjusted except the variable itself

We explored whether the CDAI–depression association differed by subgroups. As shown in Fig. 4, stratified analyses identified significant interactions by race/ethnicity (*P*-interaction = 0.014) and smoking status (*P*-interaction = 0.0007). Specifically, the inverse association was strongest among Non-Hispanic Black women (β = −0.19, 95% CI: −0.31, − 0.07) and never smokers (β = −0.07, 95% CI: −0.14, − 0.00). The interaction *P*-values for age, marital status, BMI, WBC count, or pregnancy status are non-significant (all *P*-interaction > 0.05).

## Discussion

In this cross-sectional study of pregnant and postpartum women in the United States, we found a clear association between overall dietary antioxidant capacity (measured by CDAI) and depressive symptomatology. However, this association was not linear; it was characterized by a threshold effect. Specifically, among women with very low antioxidant diets (CDAI below approximately − 2.75), higher dietary antioxidant intake was strongly associated with lower PHQ-9 depression scores. In this range, even modest improvements in dietary antioxidant intake (e.g., consuming more fruits, vegetables, and other antioxidant-rich foods) were linked to a meaningful reduction in depressive symptoms. By contrast, for the majority of women who had at least moderate antioxidant intake (CDAI above the threshold), there was no significant further relationship between CDAI and depression—their depressive symptom levels were relatively insensitive to additional increases in antioxidant consumption. To our knowledge, this is the first study to demonstrate such a nonlinear association between diet-based antioxidant capacity and perinatal depression. These findings highlight the potential importance of adequate nutrition in mental health during pregnancy and the postpartum period, while also indicating that simply “more is better” may not apply once a nutrient threshold is met.

Our results are broadly consistent with findings from general adult populations, but with some interesting distinctions. Zhao et al. analyzed adults across NHANES 2007–2018 and reported a negative nonlinear association between CDAI and depression prevalence in the overall population [21]. They identified an inflection point at CDAI ≈ 0.16 in the logistic regression context; below that, each unit increase in CDAI was associated with about a 30% reduction in depression risk, whereas beyond that point the marginal benefit was smaller (about 11% reduction per unit). Our study in perinatal women similarly found a diminishing benefit of antioxidants at higher intakes, reinforcing the idea of a saturation effect. However, the threshold in our analysis (CDAI ~ − 2.75) was substantially lower than that observed by Zhao et al. in the general adult sample. This discrepancy could be due to differences in the population characteristics and dietary patterns. Pregnant and postpartum women often have overall better nutrition and may take prenatal supplements, leading to generally higher CDAI values; thus, only the lower tail of our sample (the most nutritionally deprived 10%) had CDAI values low enough to reveal the steep gradient with depression. In contrast, the general adult population includes more individuals with poor diets (hence a threshold near the population average CDAI). Another possibility is that pregnancy itself, with its increased oxidative stress and nutritional demands, might shift the point at which antioxidant intake becomes sufficient. It may require a higher intake to counterbalance the oxidative stress of pregnancy/postpartum, meaning that only when CDAI is well above the typical non-pregnant average do additional antioxidants cease to add benefit. This is speculative, but it aligns with the notion that nutritional needs are elevated in pregnancy.

Our findings also align with emerging evidence specifically linking dietary pattern to perinatal depression. A prospective cohort from Greece by Apostolaki et al. demonstrated that women in the highest tertile of a “health-conscious” dietary pattern during pregnancy had significantly lower postpartum depression scores and roughly half the risk of high depressive symptom levels compared to those in the lowest tertile [18]. That dietary pattern was characterized by high intake of vegetables, fruits, legumes, nuts, dairy, fish, and olive oil—foods rich in antioxidants and anti-inflammatory nutrients. This is consistent with our observation that inadequate intake of such foods (reflected in low CDAI) is associated with worse mood outcomes. Another recent study by Tabaeifard et al. focused on an Oxidative Balance Score (OBS) (which incorporates both antioxidant and pro-oxidant factors) in pregnant Iranian women [8]. They found that women with higher OBS (indicating higher antioxidants and lower pro-oxidants) during pregnancy had a 69% lower risk of developing postpartum depression compared to those with low OBS. While OBS is a broader index, the results support the idea that an oxidative stress-related mechanism may underlie part of the risk for perinatal depression and that diets with greater antioxidant properties are protective. Furthermore, observational studies have noted that greater consumption of antioxidant-rich foods (like fruits, leafy greens, and vitamin C-rich produce) is associated with lower odds of postpartum depression, and even micronutrient supplementation has shown promise—for example, a randomized trial found that selenium supplementation during pregnancy significantly reduced the incidence of postpartum depressive symptoms. Selenium is one of the key components of CDAI, as it plays a crucial role in antioxidant defense (via selenoproteins like glutathione peroxidase). The positive impact of selenium supplementation on mood provides a causal hint that correcting antioxidant deficiencies can improve perinatal mental health.

The observed interaction between CDAI and race/ethnicity adds an important layer of complexity to our findings. Notably, the inverse association between dietary antioxidant intake and depressive symptoms was most pronounced among Non-Hispanic Black women, whereas no significant associations were observed in other racial/ethnic groups. This heterogeneity may be attributed to several interconnected factors. First, racial and ethnic minorities in the U.S., particularly Non-Hispanic Black populations, face disproportionate exposure to structural barriers such as residential segregation, limited access to affordable nutrient-dense foods, and higher levels of psychosocial stress related to discrimination and socioeconomic disadvantage [26, 27]. These factors may simultaneously increase vulnerability to both poor diet and depression, potentially amplifying the protective effects of improved antioxidant intake in this subgroup. Second, cultural differences in dietary patterns and food preparation practices may influence the composition and bioavailability of antioxidants, thereby modifying the relationship between CDAI and mental health outcomes [28]. Third, the observed effect modification may reflect unmeasured confounding by factors such as food insecurity, neighborhood environment, or healthcare access, which are differentially distributed across racial/ethnic groups. Future research should move beyond simple demographic adjustments and incorporate multilevel frameworks that account for structural determinants of health, including racism, discrimination, and neighborhood-level resources, to better understand the mechanisms underlying these racial/ethnic differences.

The biological plausibility of our findings is strengthened by what is known about oxidative stress and inflammation in depression [9]. Pregnancy is a state of heightened oxidative stress, which tends to peak in late gestation and the early postpartum period due to factors like increased metabolic activity, mitochondrial generation of free radicals, and, during delivery, sudden changes in oxygenation and possible tissue injury. If a woman’s diet is deficient in antioxidants, she may not adequately neutralize the excess free radicals, leading to cellular damage and promotion of inflammatory pathways. Chronic, low-grade inflammation and oxidative damage in the central nervous system have been implicated in the pathogenesis of depression [29]. In our analysis, we saw that high WBC count was associated with higher depressive symptoms, consistent with this theory. Dietary antioxidants such as vitamins C and E can directly scavenge reactive oxygen species, whereas others like zinc and selenium [30] are cofactors for antioxidant enzymes and support immune function. By bolstering the body’s antioxidant defenses, a sufficient intake of these nutrients might reduce neuro-inflammation and oxidative damage to neural tissues, thus potentially ameliorating or preventing depressive symptoms. Notably, one could consider that beyond a certain intake level, antioxidant systems are saturated—e.g., enzymes are at full capacity and cellular redox status is balanced—so additional antioxidants will simply be excreted or not utilized, yielding no extra benefit to mood. This concept fits with our observed plateau: once the “antioxidant need” is met (which most women in our sample achieved), other factors likely drive depression risk, such as psychosocial stress, genetics, or hormonal changes, and adding more antioxidants doesn’t further reduce risk.

Our study has several strengths. It leverages a large, multi-year national dataset with standardized measures, allowing for greater generalizability and sufficient power to detect nonlinear effects. The use of CDAI as an exposure is innovative in the perinatal context, as it captures the overall antioxidant capacity of the diet. We were able to adjust for numerous confounders, including socio-economic and lifestyle factors, which improves confidence that the observed association is not spurious. Additionally, we performed extensive sensitivity analyses to test the robustness of the findings. The consistency of the threshold effect across these lends credibility to the result. Our focus on pregnant and postpartum women addresses a gap in research, as most previous studies of diet and depression looked at general or older populations [31]. These results can inform hypotheses for future intervention trials specifically in maternal populations.

However, certain limitations must be acknowledged. First, the study design is cross-sectional. We measured diet and depressive symptoms at the same time; thus, we cannot establish causation or the direction of the relationship. It is possible that women with depression symptoms at the time of the survey changed their eating habits (e.g., ate less or differently), which could lower their CDAI. While we attempted to mitigate this by controlling for various covariates, unmeasured factors related to both diet and depression (like overall health consciousness or unreported psychosocial stress) could confound the results.

Second, this study did not apply NHANES sampling weights in the primary analyses. While we focused on examining associations between CDAI and depressive symptoms rather than producing nationally representative prevalence estimates, the lack of weighting may limit the generalizability of our findings to the broader U.S. perinatal population. Nonetheless, our approach yields internally valid estimates of associations, and the consistency of our results across multiple sensitivity analyses supports the robustness of the observed relationships. Future studies using NHANES data should consider applying appropriate sampling weights to assess the generalizability of similar associations.

Third, dietary intake data from 24-hour recalls have inherent measurement error and day-to-day variability. Although we used two recalls when available and calculated a composite index to reduce measurement noise, there will still be some misclassification of true usual antioxidant intake. This non-differential measurement error tends to attenuate associations, meaning our estimates may actually underestimate the true diet-depression relationship.

Fourth, the CDAI used in this study captures only dietary antioxidants and does not incorporate pro-oxidant dietary components (e.g., processed meats, added sugars, ultra-processed foods) or other dietary factors linked to depression, such as protein, fiber, omega-3 fatty acids, pro-inflammatory dietary patterns, or gut microbiome-related factors [32, 33]. As such, it may not fully reflect the net oxidative balance or overall dietary quality of individuals. A person with a high CDAI could still have a predominantly pro-oxidant dietary pattern, while another with a moderate CDAI might consume an overall healthy diet rich in fibers and polyphenols—factors not captured by the CDAI. Future studies should consider employing more comprehensive indices (e.g., oxidative balance scores) that integrate both antioxidant and pro-oxidant exposures, as well as other dietary components relevant to mental health.

Fifth, our CDAI did not include contributions from nutritional supplements. Many perinatal women take prenatal vitamins containing antioxidants (e.g., vitamins C, E, A, and zinc). If a woman had a low dietary intake but was compensating with a supplement, our analysis might classify her as low CDAI even though her total antioxidant supply was higher. This non-differential misclassification would likely bias the association toward the null, meaning our estimates may actually underestimate the true diet–depression relationship. The fact that we still observed a strong association at low CDAI suggests that those truly deficient in diet (and possibly not supplementing adequately) are driving the effect. Nonetheless, future studies could improve on this by constructing a ‘total antioxidant intake index’ that includes supplements, or by using objective biomarkers of antioxidant status (e.g., serum nutrient levels).

Sixth, although we adjusted for white blood cell count as a proxy for inflammation, this measure is more indicative of acute infection rather than chronic low-grade inflammation. C-reactive protein (CRP) would have been a more specific marker of chronic inflammation relevant to depression pathophysiology [34]. However, CRP data were not available across all NHANES cycles included in our study (2005–2018). Future studies should consider incorporating CRP or other inflammatory biomarkers (e.g., IL-6, TNF-α) to better elucidate the potential mediating role of inflammation in the relationship between dietary antioxidant intake and perinatal depressive symptoms.

Finally, although we adjusted for poverty income ratio as a proxy for socioeconomic status, this measure does not capture the dynamic and multidimensional nature of food insecurity. Food insecurity—defined as limited or uncertain access to adequate and safe food—has been independently associated with both poor dietary quality and increased risk of perinatal depression [35, 36]. Notably, food insecurity measures are not consistently available across all NHANES cycles included in our study (2005–2018). Unlike static income-based measures, food insecurity reflects material deprivation, coping strategies, and cyclical resource constraints that may more directly influence nutritional intake and mental health. Our inability to account for food insecurity may have led to residual confounding, particularly given its disproportionate burden on racial/ethnic minority and low-income populations [27]. Importantly, if food insecurity is associated with both lower CDAI (due to reduced access to antioxidant-rich foods) and higher depressive symptoms, the true association between dietary antioxidants and perinatal depression may be overestimated or underestimated depending on the complex interplay of these factors. Future studies should incorporate validated food security assessments (e.g., USDA Household Food Security Survey Module) to better disentangle the roles of income, food access, and nutritional quality in shaping perinatal mental health outcomes.

In addition, residual confounding from other unmeasured psychosocial factors remains possible. We did not have data on history of depression prior to pregnancy—a factor strongly associated with both perinatal depression risk and, potentially, dietary patterns. If pre-pregnancy depression leads to both poorer diet (lower CDAI) and higher perinatal depressive symptoms, then our failure to adjust for it may have led to an overestimation of the true association. Future prospective cohort studies that enroll women before pregnancy or in early pregnancy are needed to adequately control for pre-pregnancy mental health history and to establish temporal relationships. Similarly, we did not adjust for social support or physical activity, both of which are linked to diet and mental health [37–40]. For instance, if women with low CDAI also had less social support or were less physically active, part of their elevated depression risk could be attributable to these factors rather than diet al.one. Unfortunately, NHANES does not systematically collect detailed data on these variables across all cycles. Future prospective studies should incorporate validated measures of social support and physical activity to disentangle their independent effects from diet on perinatal mental health.

In light of these results, several implications emerge. For clinicians and public health professionals in maternal care, nutrition should be considered as one aspect of holistic prenatal and postpartum care that could influence mental health. Screening for depressive symptoms is now recommended as part of routine obstetric care [41]; our findings suggest that it may also be useful to inquire about dietary habits or ensure nutritional adequacy, especially for women who screen positive for depression or have other risk factors. Women with very poor diets might benefit from nutritional counseling or dietitian referral as part of their depression management plan. Ensuring adequate intake of fruits, vegetables, and prenatal vitamins (as needed) might help improve mood or at least remove one potential contributor to depression. It is important to clarify that diet is not a sole solution—perinatal depression is multifactorial, and evidence-based treatments include psychotherapy and, where appropriate, antidepressant medication [1]. However, dietary improvement is a low-risk intervention that could complement other treatments and provide overall health benefits for mother and baby. The notion of using “food as medicine” is attractive; trials of dietary interventions in general depressed populations (such as Mediterranean diet interventions) have shown promising reductions in depressive symptoms [7, 42]. Similar trials in pregnant/postpartum women are relatively scarce but warranted. Our results encourage such trials—for example, a randomized trial providing antioxidant-rich whole foods or supplements to nutritionally at-risk pregnant women could test whether it reduces the incidence of postpartum depression.

In summary, this study provides evidence that adequate—not excessive—antioxidant intake may be sufficient for mental health benefits during the perinatal period. This “more is not always better” finding has important implications for patient education in an era of widespread wellness marketing that often promotes high-dose antioxidant supplements. From a clinical perspective, dietary quality can be viewed as a modifiable, accessible target—a “low-hanging fruit”—for supporting mental health in at-risk perinatal populations.

From a research perspective, future studies should aim to replicate these findings prospectively and delve deeper into mechanisms. A longitudinal study following women from early pregnancy through postpartum, with repeated dietary assessments and depression evaluations, would help establish temporal relationships and possibly causality. Biomarkers of oxidative stress and antioxidant status (e.g., blood levels of vitamins, glutathione, and inflammatory cytokines) could be measured to provide objective correlates to self-reported diet and depression outcomes. It would also be valuable to examine if particular nutrients drive the association—for instance, perhaps most of the effect comes from vitamin C and selenium, or from a combination—which could inform targeted nutritional recommendations. Additionally, one might explore interactions: do certain women benefit more from high-antioxidant diets? For example, genetic differences in antioxidant enzyme function or differences in baseline inflammation might modify the impact of diet.

## Conclusion

In conclusion, this study provides evidence that composite dietary antioxidant index is inversely associated with depressive symptoms in pregnant and postpartum women, particularly when antioxidant intake is very low. Beyond a threshold level of intake, additional antioxidants do not appear to further reduce depression risk, suggesting the relationship is one of meeting a nutritional adequacy for mental health rather than a simple linear dose-response. There was an interaction between CDAI and smoking status, as well as race/ethnicity. The inverse association was more pronounced among women who had never smoked. These findings contribute to a growing literature on the role of nutrition in perinatal mental health and underscore the importance of a balanced, nutrient-rich diet during pregnancy and after childbirth. 

## Supplementary Information

Supplementary Material 1: Supplementary Material 1: Table S1 Availability of 24‑hour dietary recalls in NHANES cycles (2005–2018) among the overall NHANES population and the study sample of perinatal women. Supplementary Material 2: Fig. S1 The association between Composite dietary antioxidant index and depressive symptoms (PHQ-9 total score ≥5). Supplementary Material 3: Fig. S2 The association between Composite dietary antioxidant index and depressive symptoms (PHQ-9 total score ≥15). Supplementary Material 4: Table S2 Threshold effect analysis of Composite dietary antioxidant index on depressive symptoms (PHQ-9 total score ≥5). Supplementary Material 5: Table S3 Threshold effect analysis of Composite dietary antioxidant index on depressive symptoms (PHQ-9 total score ≥15). Supplementary Material 6: Fig. S3 The association between Composite dietary antioxidant index and PHQ-9 total scores, with additional adjustment for C-reactive protein. Supplementary Material 7: Fig. S4 The association between Composite dietary antioxidant index and Depression (PHQ-9 total score ≥10), with additional adjustment for C-reactive protein.

## Data Availability

The datasets presented in this study can be found in online repositories. The names of the repository/repositories and accession number(s) can be found below: All data are available in the NHANES database (www.cdc.gov/nchs/nhanes).
