# Supplementary material for: Associations between composite dietary antioxidant index and depressive symptoms among pregnant and postpartum women
Source: BMC Pregnancy Childbirth. 2026 Apr 10;26:547. doi: 10.1186/s12884-026-09047-8 (PMC13188579; doi:10.1186/s12884-026-09047-8)

**Supplementary material**

Supplementary Material 1: Table S1 Availability of 24‑hour dietary recalls in NHANES cycles (2005–2018) among the overall NHANES population and the study sample of perinatal women.

Supplementary Material 2: Fig. S1 The association between Composite dietary antioxidant index and depressive symptoms (PHQ-9 total score ≥5).

Supplementary Material 3: Fig. S2 The association between Composite dietary antioxidant index and depressive symptoms (PHQ-9 total score ≥15).

Supplementary Material 4: Table S2 Threshold effect analysis of Composite dietary antioxidant index on depressive symptoms (PHQ-9 total score ≥5).

Supplementary Material 5: Table S3 Threshold effect analysis of Composite dietary antioxidant index on depressive symptoms (PHQ-9 total score ≥15).

Supplementary Material 6: Fig. S3 The association between Composite dietary antioxidant index and PHQ-9 total scores, with additional adjustment for C-reactive protein.

Supplementary Material 7: Fig. S4 The association between Composite dietary antioxidant index and Depression (PHQ-9 total score ≥10), with additional adjustment for C-reactive protein.

**Table S1** Availability of 24‑hour dietary recalls in NHANES cycles (2005–2018) among the overall NHANES population and the study sample of perinatal women.

| **NHANES cycle** | **Overall NHANES population** | | | | **Study sample (perinatal women)** | | | |
| --- | --- | --- | --- | --- | --- | --- | --- | --- |
|  | **N** | **CDAI1** | **CDAI2** | **n (%)** | **N** | **CDAI1** | **CDAI2** | **n (%)** |
| 2005–2006 | 10,348 | 9,169 | 8,264 | 905 (9.87%) | 271 | 271 | 271 | 0 (0%) |
| 2007–2008 | 10,149 | 9,118 | 7,715 | 1,403 (15.39%) | 136 | 136 | 136 | 0 (0%) |
| 2009–2010 | 10,537 | 9,623 | 8,288 | 1,335 (13.87%) | 150 | 150 | 150 | 0 (0%) |
| 2011–2012 | 9,756 | 8,389 | 7,496 | 893 (10.64%) | 102 | 102 | 102 | 0 (0%) |
| 2013–2014 | 10,175 | 8,531 | 7,453 | 1,078 (12.64%) | 161 | 161 | 161 | 0 (0%) |
| 2015–2016 | 9,971 | 8,327 | 6,875 | 1,452 (17.44%) | 141 | 141 | 141 | 0 (0%) |
| 2017–2018 | 9,254 | 7,484 | 6,502 | 982 (13.12%) | 132 | 132 | 132 | 0 (0%) |
| Total | 70,190 | 60,641 | 52,593 | 8,048 (13.27%) | 1,093 | 1,093 | 1,093 | 0 (0%) |

CDAI1, number of participants with non‑missing values for the first 24‑hour dietary recall; CDAI2, number of participants with non‑missing values for the second 24‑hour dietary recall; n (%), participants with only one recall.

**Fig. S1** The association between Composite dietary antioxidant index and depressive symptoms (PHQ-9 total score ≥5). Models were adjusted for age, ethnicity, poverty income ratio, marital status, white blood cell count, body mass index, smoking status, and alcohol use


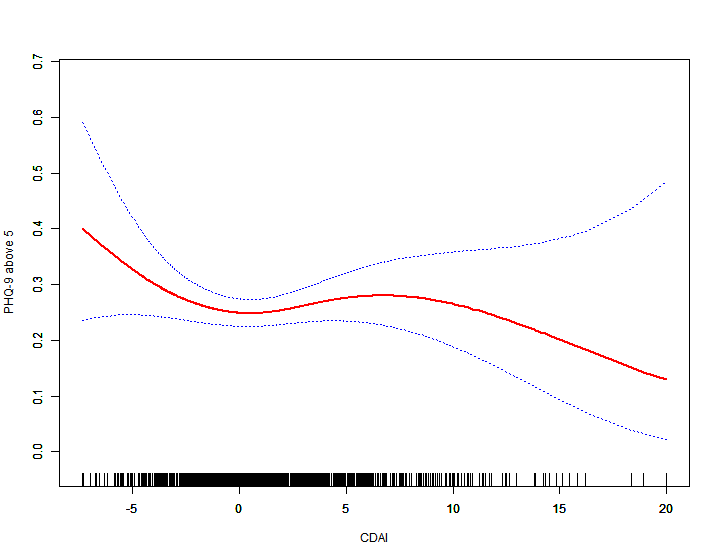


**Fig. S2** The association between Composite dietary antioxidant index and depressive symptoms (PHQ-9 total score ≥15). Models were adjusted for age, ethnicity, poverty income ratio, marital status, white blood cell count, body mass index, smoking status, and alcohol use


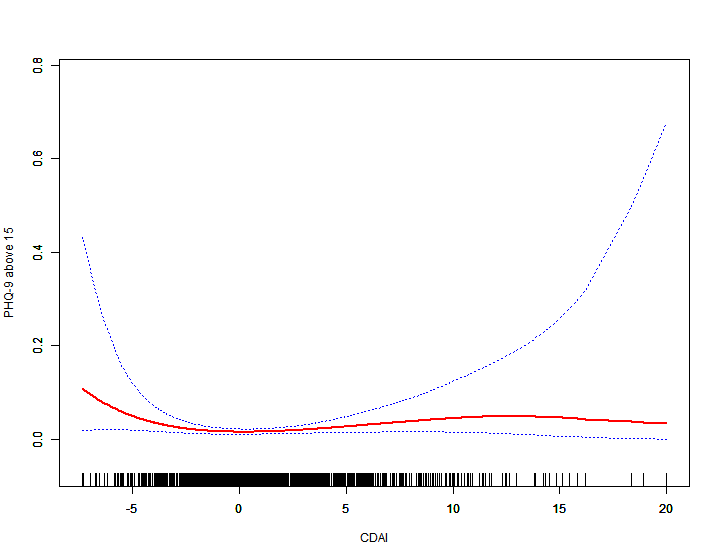


**Table S2** Threshold effect analysis of Composite dietary antioxidant index on depressive symptoms (PHQ-9 total score ≥5).

|  | **OR (95 % CI)** | ***P*-value** |
| --- | --- | --- |
| Fitting by standard linear model | 0.99 (0.95, 1.02) | 0.5000 |
| Fitting by two-piecewise linear model |  |  |
| Inflection point | -2.75 |  |
| ≤ -2.75 | 0.77 (0.61, 0.99) | 0.0406 |
| > -2.75 | 1.01 (0.97, 1.05) | 0.7844 |
| Log-likelihood ratio | 0.05 |  |

Models were adjusted for age, ethnicity, poverty income ratio, marital status, white blood cell count, body mass index, smoking status, and alcohol use.

**Table S3** Threshold effect analysis of Composite dietary antioxidant index on depressive symptoms (PHQ-9 total score ≥15).

|  | **OR (95 % CI)** | ***P*-value** |
| --- | --- | --- |
| Fitting by standard linear model | 1.02 (0.92, 1.13) | 0.7559 |
| Fitting by two-piecewise linear model |  |  |
| Inflection point | -2.75 |  |
| ≤ -2.75 | 0.49 (0.30, 0.79) | 0.0033 |
| > -2.75 | 1.09 (0.98, 1.21) | 0.1003 |
| Log-likelihood ratio | 0.006 |  |

Models were adjusted for age, ethnicity, poverty income ratio, marital status, white blood cell count, body mass index, smoking status, and alcohol use.

**Fig. S3** The association between Composite dietary antioxidant index and PHQ-9 total scores, with additional adjustment for C-reactive protein. Models were adjusted for age, ethnicity, poverty income ratio, marital status, white blood cell count, body mass index, smoking status alcohol use, and C-reactive protein


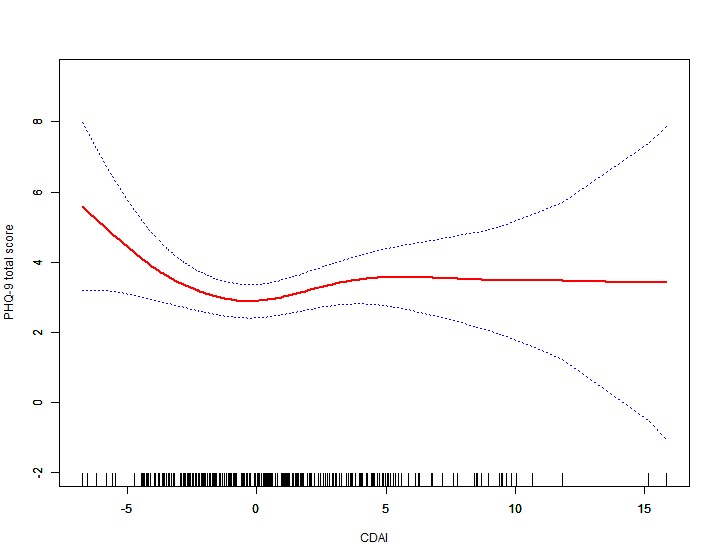


**Fig. S4** The association between Composite dietary antioxidant index and Depression (PHQ-9 total score ≥10), with additional adjustment for C-reactive protein. Models were adjusted for age, ethnicity, poverty income ratio, marital status, white blood cell count, body mass index, smoking status alcohol use, and C-reactive protein


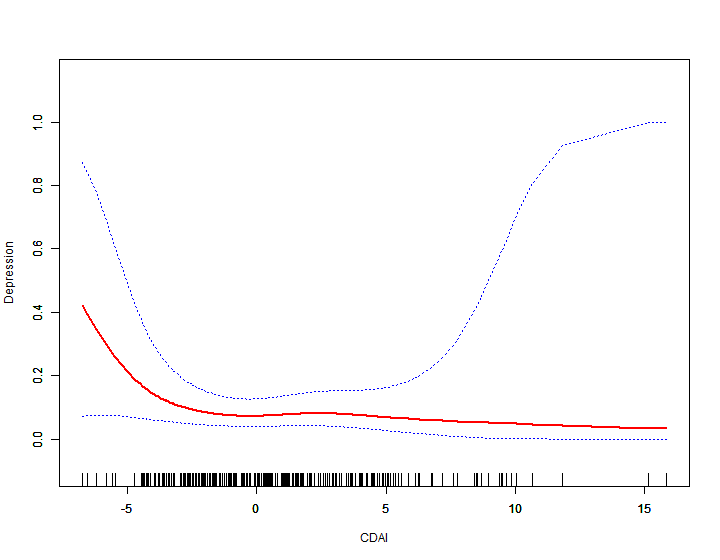

Supplement: Supplementary file 1 — Supplementary Material 1: Supplementary Material 1: Table S1 Availability of 24‑hour dietary recalls in NHANES cycles (2005–2018) among the overall NHANES population and the study sample of perinatal women. Supplementary Material 2: Fig. S1 The association between Composite dietary antioxidant index and depressive symptoms (PHQ-9 total score ≥5). Supplementary Material 3: Fig. S2 The association between Composite dietary antioxidant index and depressive symptoms (PHQ-9 total score ≥15). Supplementary Material 4: Table S2 Threshold effect analysis of Composite dietary antioxidant index on depressive symptoms (PHQ-9 total score ≥5). Supplementary Material 5: Table S3 Threshold effect analysis of Composite dietary antioxidant index on depressive symptoms (PHQ-9 total score ≥15). Supplementary Material 6: Fig. S3 The association between Composite dietary antioxidant index and PHQ-9 total scores, with additional adjustment for C-reactive protein. Supplementary Material 7: Fig. S4 The association between Composite dietary antioxidant index and Depression (PHQ-9 total score ≥10), with additional adjustment for C-reactive protein. [file 12884_2026_9047_MOESM1_ESM.docx]
